# Biological characterization of genetically modified trees (GMTs)

**DOI:** 10.1186/1753-6561-5-S7-O58

**Published:** 2011-09-13

**Authors:** Gilles Pilate, Hely Haggman, Isabel Allona, Lucian Curtu, Terezia Salaj, Ove Nilsson, Matthias Fladung, Cristina Vettori

**Affiliations:** 1 INRA, UR588 Amélioration, Génétique, et Physiologie Forestières, F-45075 Orléans cedex 2, France; 2 University of Oulu, Department of Biology, P.O. Box 3000, FI-90014 Oulu, Finland; 3 Centro de Biotecnologia y Genomica de Plantas (CBGP-UPM/INIA), Campus de Montegancedo, UPM, 28223 Madrid, Spain; 4 Transilvania University Brasov, Faculty of Forest Sciences, 1 Sirul Beethoven, Brasov, Romania; 5 Institute of Plant Genetics and Biotechnology, Slovak Academy of Sciences, Akademická 2, P. O. Box 39A, 950 07 Nitra, Slovakia; 6 Department of Forest Genetics and Plant Physiology, SLU; 7 Johann Heinrich von Thünen Institute (vTI) Institute for Forest Genetics Sieker Landstr. 2, D-22927 Großhansdorf, Germany; 8 Plant Genetics Institute, National Research Council, Via Madonna del Piano 10, 50019 Sesto, Fiorentino (FI), Italia

## 

In the last 20 years, a large number of GMTs have been generated, characterized and field tested to evaluate their agronomic behaviour and their potential performance for technological applications.

Therefore, important knowledge has been gained from these studies on the potential of GMTs whereas biosafety concerns have been raised on the plantation of transgenic trees in the field. The European Cooperation in Science and Technology (COST) action FP0905 on the biosafety of forest transgenic trees aims to help forthe building and implementation of EU policy directives to ensure a safe development and practical use of GMTs in the future. This Action is expected to generate important benefits thanks to the experience gained by the research community, to provide general information on these studies as well as to address public concerns about biosafety issues raised by transgenic tree plantations, and finally to help for the establishment and implementation of EU policy directives in the view of a future deployment of GMTs cultivation in Europe. Within this action, the objective of the working group 1 (WG1) is to gather the existing knowledge on genetically modified forest trees (GMTs) including both field trials and greenhouse experiments. More specifically, the action of WG1 is focused on the compilation of:

- Data from literature and methods developed on gene flow and containment strategies

- Data available on the traits targeted and the function of the gene introduced, on their intended and eventually unintended effects

- Data on host species characteristics especially related to biosafety issues such as their biological cycle, their way of pollination and the characteristics of pollen and seed dispersal.

This information will be collected from published papers in books and reviews as well as from reports from different agencies available on the web. Moreover, additional data will hopefully be gained through the responses of expert scientists from public or private institutions to an ongoing survey accessible at the website of the COST Action FP0905: [http://www.cost-action-fp0905.eu/index.php?option=com_kunena&Itemid=74&func=view&catid=4&id=11]

The information collected will be used to:

- 1) establish a list of protocols for the control of transgene flow and gene containment.

- 2) establish the present state of the art in gene targeting strategies for site specific integration of transgenes.

- 3) get an appraisal on the types of construct and genes inserted in GMTs in the past; in the present and in the future.

This information will be compiled in a database that will be made available to the scientific community as well as to the European policy makers.

The results of this work will enable the participants and end-users to get a clear factual overview of the global status of GMTs and especially the status of Europe compared to non-European countries. Furthermore, the information gathered will also be useful for assessment of environmental impacts of GMTs that is the object addressed in the WG2 of the COST Action FP0905.

